# Drug repositioning for psychiatric and neurological disorders through a network medicine approach

**DOI:** 10.1038/s41398-020-0827-5

**Published:** 2020-05-12

**Authors:** Thomaz Lüscher Dias, Viviane Schuch, Patrícia Cristina Baleeiro Beltrão-Braga, Daniel Martins-de-Souza, Helena Paula Brentani, Glória Regina Franco, Helder Imoto Nakaya

**Affiliations:** 1 Departament of Biochemistry and Immunology, Institute of Biological Sciences, Federal University of Minas Gerais, Belo Horizonte, Brazil; 2 Department of Clinical and Toxicological Analyses, School of Pharmaceutical Sciences, University of São Paulo, São Paulo, Brazil; 3 Department of Microbiology, Institute of Biomedical Sciences, University of São Paulo, São Paulo, Brazil; 4 Scientific Platform Pasteur USP, São Paulo, Brazil; 5 Laboratory of Neuroproteomics, Department of Biochemistry and Tissue Biology, Institute of Biology, University of Campinas, Campinas, Brazil; 6 Instituto Nacional de Biomarcadores em Neuropsiquiatria, Conselho Nacional de Desenvolvimento Científico e Tecnológico, São Paulo, Brazil; 7 Experimental Medicine Research Cluster (EMRC), University of Campinas, Campinas, Brazil; 8 D’Or Institute of Reasearch and Education (IDOR), São Paulo, Brazil; 9 Instituto de Psiquiatria, Hospital das Clínicas HCFMUSP, Faculdade de Medicina, Universidade de São Paulo, São Paulo, Brazil; 10 National Institute of Developmental Psychiatry for Children and Adolescents (INPD), São Paulo, Brazil

**Keywords:** Psychiatric disorders, Pharmacology

## Abstract

Psychiatric and neurological disorders (PNDs) affect millions worldwide and only a few drugs achieve complete therapeutic success in the treatment of these disorders. Due to the high cost of developing novel drugs, drug repositioning represents a promising alternative method of treatment. In this manuscript, we used a network medicine approach to investigate the molecular characteristics of PNDs and identify novel drug candidates for repositioning. Using IBM Watson for Drug Discovery, a powerful machine learning text-mining application, we built knowledge networks containing connections between PNDs and genes or drugs mentioned in the scientific literature published in the past 50 years. This approach revealed several drugs that target key PND-related genes, which have never been used to treat these disorders to date. We validate our framework by detecting drugs that have been undergoing clinical trial for treating some of the PNDs, but have no published results in their support. Our data provides comprehensive insights into the molecular pathology of PNDs and offers promising drug repositioning candidates for follow-up trials.

## Introduction

Psychiatric and neurological disorders (PNDs) represent a burden for public health. The World Health Organization estimates that at least 450 million people suffer from PNDs (ref. ^1^). Depression (322 million affected)^2^, bipolar disorder (60 million)^1^, schizophrenia (23 million)^3^, dementia and Alzheimer’s disease (50 million)^1^, and anxiety (260 million)^2^ are the most prevalent PNDs in the world. Autism spectrum disorders (1 in 59 children)^4^ and PNDs, such as Huntington’s disease (5–7 in 100,000 affected)^1^, and Parkinson’s disease (1–4% of all elderly people)^1^ are also of great concern.

Five major classes of drugs are used to treat PNDs: antidepressants, antipsychotics, anxiolytics, mood stabilizers, and stimulants. However, disease remission is not always achieved^5,6^. This stems from an incomplete knowledge of the molecular mechanisms of both PNDs (ref. ^7^) and the psychiatric drugs^8^. In addition, PNDs share several clinical and genetic components^9^, which makes the precise treatment and a subsequent targeted drug development more challenging^10^. Specifically, drug repositioning, which relies on testing drugs already in use for a disease to treat another illness based on the shared molecular pathology of both^11^, may be applied to treat PNDs (ref. ^12^).

Network medicine^13^ is an emerging field that combines systems biology and network science to understand how genes interact in disease and health. For PNDs, co-expression networks^14–16^ and genome-wide association studies^9,17,18^ have unraveled molecular mechanisms and genomic variations related to these disorders. Many more small-scale studies have investigated the roles of specific genes in PNDs. The daunting task, now, is to make sense of all the published data, stored in millions of research papers, that describe the interplay among genes, drugs, and other variables in the development and outcomes of PNDs.

Here, we used a network medicine approach to dissect the molecular mechanisms of PNDs and identify novel drug candidates for repositioning. Using IBM Watson for Drug Discovery (WDD), a machine learning text-mining application, we built knowledge networks containing connections between PNDs and genes or drugs mentioned in the published scientific literature in the past 50 years. We found classic and potentially unexplored pathways associated to PNDs. We also identified several drugs that target key PND-related genes that have never been used to treat these disorders previously. Validating our approach, some of these drugs are currently being tested to treat PNDs in clinical trials, with no previously published results. Our data provides comprehensive insight into the molecular pathology of PNDs and offers promising drug repositioning candidates for follow-up trials.

## Materials and methods

### Construction of the knowledge networks

We used the IBM WDD, an online tool, to perform queries for major PNDs: Alzheimer’s disease, dementia, anxiety, depression, Huntington’s disease, Parkinson’s disease, schizophrenia, bipolar disorder, and autism. We performed two independent searches using WDD: one for genes associated with PNDs (gene–PND) and another for drugs associated with PNDs (drug–PND). WDD detects associations in original papers and reviews from PMC Open Access (full text), Pubmed (abstracts), and patents. Relationships are detected by a natural language-processing algorithm when two entities of interest (gene, disease, or drug) are present in the same sentence of a document and are connected through a verb or preposition. WDD gives relationships a confidence score (0–100%) based on the number of documents supporting the connection and on link meaningfulness. Only relations with at least two documents of evidence and a confidence score >50% were maintained in our study. Searches were performed from July to August of the year 2018.

### Network analysis

The Louvain^19^ method was used to detect modules of highly connected genes or drugs. For each pair of PNDs in the gene and drug networks, we performed Fisher’s exact test to calculate the significance of the overlap of genes or drugs in PNDs. Fisher’s exact test *p*-values (<0.01) were considered significant. Results were presented as −log_10_*p*-value.

### Functional gene enrichment

Genes from modules in the gene–PND network and those exclusively associated with each PND were submitted for functional gene enrichment using *enrichR* (ref. ^20^). Enrichment was performed against the Gene Ontology Biological Process and the KEGG databases. The enriched terms with an *enrichR* combined score of at least 20 and *p* < 0.01 were retained, and the most enriched terms were used to describe the results.

### Drug repositioning

Gene co-expression modules of PNDs were obtained from the Supplementary material of Gandal^15^. Co-expression modules were detected and the first principal component of the module’s expression (eigengene) was used to determine module–disease association. Module membership (*k*ME) was calculated for each gene. The *k*ME is equal to the Pearson’s correlation *R* between the expression of the gene and the eigengene of the module. Module hubs are those that have *k*ME > 0.5 in their module.

The repositioning with Open Targets^21^ database was performed using a custom R code. This script, which is available on GitHub (https://github.com/csbl-usp/OpenTargets_drug_repositioning), accesses the platform API client of Open Targets database and performs the same steps done for the drug repositioning with WDD. Only relationships with an overall association score >0.5 were utilized.

## Results

### Molecular characterization of PNDs using the scientific knowledge

**Fig. 1:**
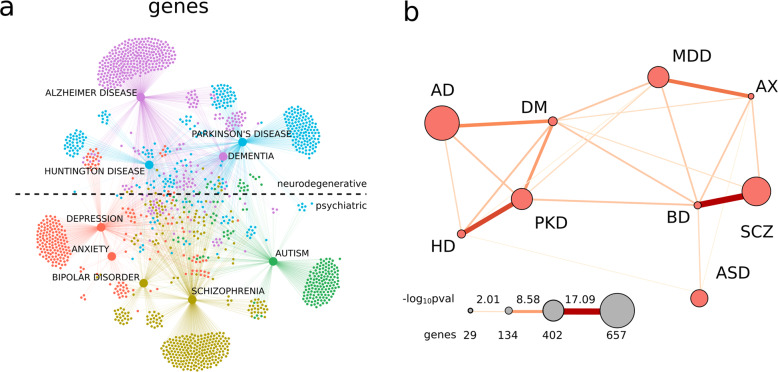
Genes shared between PNDs. **a** A knowledge network for genes colored according to Louvain-defined modules: Alzheimer’s disease (AD) and dementia (DM) (purple), Huntington’s disease (HD) and Parkinson’s disease (PKD; light blue), depression (MDD) and anxiety (AX; red), schizophrenia (SCZ) and bipolar disorder (BD; green-yellow), and autism (ASD; green). The dashed line in **a** separates neurodegenerative disorders from psychiatric disorders in the network. **b** The significance of the gene overlap between PNDs. Larger nodes represent PNDs with more genes and thicker edges represent a more significant overlap between PNDs (proportional to −log_10_*p*-value of Fisher’s exact test).

IBM WDD is a cognitive computing, artificial intelligence platform that was used to extract existing connections between genes, diseases, and drugs from millions of published documents related to the medical sciences^22^. WDD uses a dictionary created by artificial intelligence to group terms that are used conversely in the literature (e.g., gene IDs from distinct databases). It identifies relations between the searched term and other terms of interest in the literature. A natural language-processing algorithm detects these relations. We used WDD to investigate the genes and drugs that were shared among different PNDs. A total of 1588 genes and 722 drugs was identified as associated with PNDs. The network constructed with the genes associated with PNDs separated the diseases into two groups: neurodegenerative disorders (Alzheimer’s, Parkinson’s and Huntington’s diseases, and dementia) and psychiatric disorders (depression, anxiety, bipolar disorder, schizophrenia, and autism; Fig. 1a). The gene network identified five clusters of disorders with similar clinical characteristics: Alzheimer’s disease and dementia, cognition hindering neurodegenerative illnesses (Fig. 1a—purple color); Huntington’s disease and Parkinson’s disease, disorders that affect movement due to basal ganglia degeneration^23^ (Fig. 1a—light blue color); depression and anxiety, fear/threat-related disorders^24^ (Fig. 1a—red color); and schizophrenia and bipolar disorder, which share a spectrum of psychotic symptoms^25^ (Fig. 1a—brown-yellow color). Autism (Fig. 1a—green color), the only developmental PND analyzed, did not cluster with any other disease. Fisher’s exact test confirmed the division between neurodegenerative and psychiatric disorders and the associations between clinically similar disorders (Fig. 1b). The highest similarity was observed between disorders of the same group (neurodegenerative or psychiatric) and between PNDs clustered within the same modules (Fig. 1b). However, the separation between neurodegenerative and psychiatric disorders was not as evident in the PND-drug network (Supplementary Fig. 1).

### Functional gene analysis: insights into PND molecular pathology

**Fig. 2:**
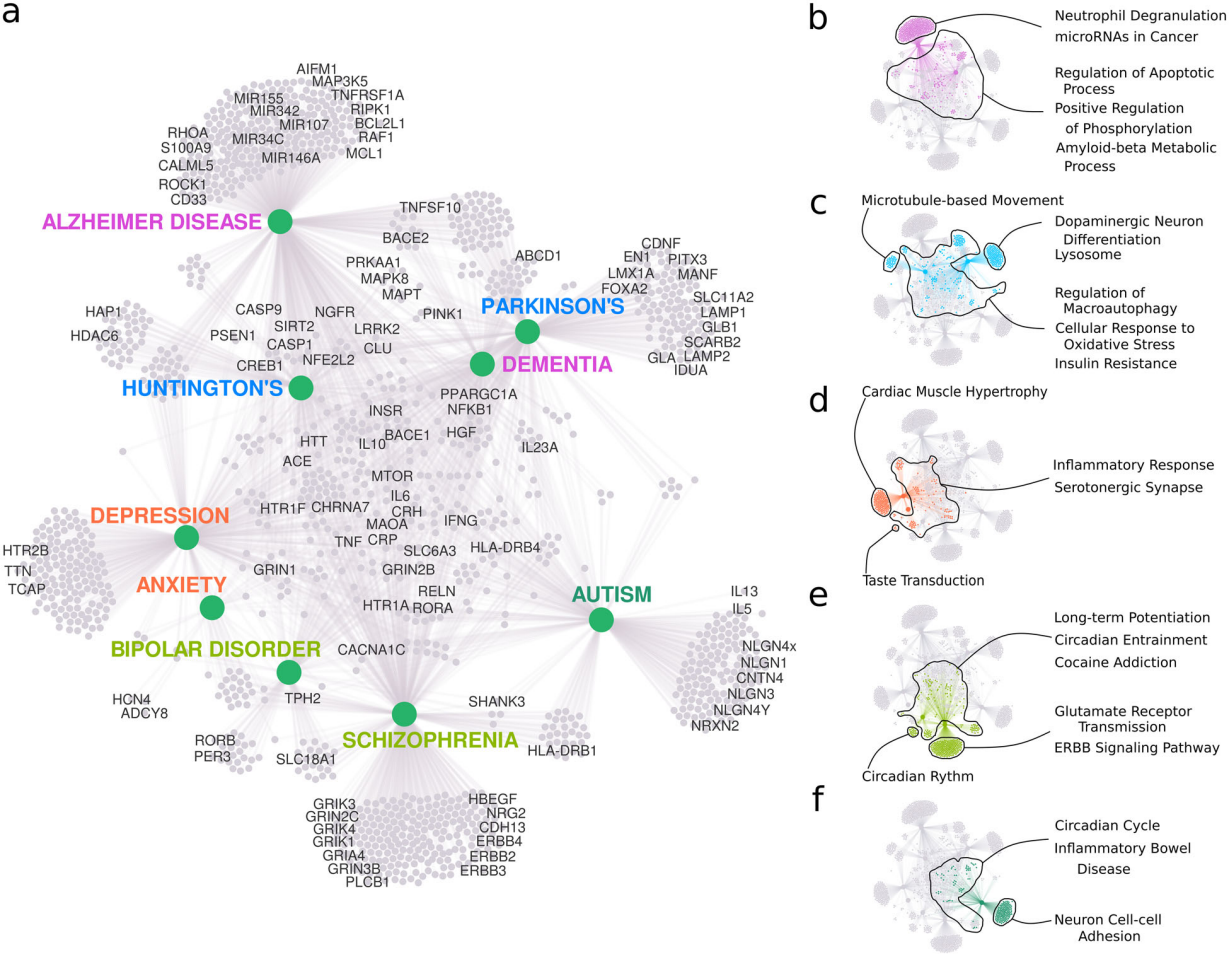
Functional analysis of genes unique to each PND and network modules. **a** A summary of genes connected to PNDs enriched for relevant biological processes (GO) and pathways (KEGG). **b**–**f** The most significant gene enrichment categories for genes in the modules: Alzheimer’s and dementia (**b**), Huntington’s disease and Parkinson’s disease (**c**), depression and anxiety (**d**), schizophrenia and bipolar disorder (**e**), and autism (**f**).

We performed functional enrichment analyses with the genes in each module and with those unique to each PND (Fig. 2a). For all the modules, we found well-known, hallmark molecular characteristics of PNDs (Fig. 2, Supplementary results). We also had potentially novel insights into the PNDs. The genes in the Alzheimer’s disease/dementia module (Fig. 2b) were strongly enriched for neutrophil degranulation (33 genes) and microRNAs in cancer (21 genes; Fig. 2b). Neutrophil phenotype alterations in Alzheimer’s disease correlate with disease progression^26^, and neutrophil depletion improves memory and slows disease progression in mice^27^. One miRNA involved in cancer and Alzheimer’s disease, miR-146a, regulates innate immune response through inflammation in both diseases^28^. Conversely, many miRNAs that stimulate proliferation in cancer seem to favor apoptosis in Alzheimer’s disease^28^. A better comprehension of how miRNAs regulate the cell cycle and the immune system can open new therapeutic opportunities for treating both cancer and Alzheimer’s disease.

Huntington’s disease and Parkinson’s disease present severe neuronal loss in the basal ganglia^23^. In this light, genes involved in insulin resistance were enriched in this module (Fig. 2c). A recent study showed that over 60% of nondiabetic Parkinson’s disease patients had insulin resistance^29^. Genes unique to Huntington’s disease were enriched for the regulation of microtubule-based movement (Fig. 2c). The huntingtin protein interacts with several cell motility proteins, including HAP1 (ref. ^30^) and HDAC6 (ref. ^31^). These interactions mediate organelle trafficking^32^, and energy production and consumption via the axonal bounding of GAPDH to synaptic vesicles^33^.

The inflammatory response was enriched in the depression/anxiety module (Fig. 2d): c-reactive protein levels have been known to predict the outcome of antidepressant treatment^34,35^ and disease severity^36^. In addition, immunomodulatory proteins were found to be altered in the postmortem brain^37^, blood, and plasma^38^ of depression patients. Genes uniquely connected to depression were enriched for striated and cardiac muscle hypertrophy (*HTR2B*, *TCAP*, and *TTN*; Fig. 2d). Anxiety shares most of its genes with depression and has only eight unique genes (Fig. 2d). Two of these, *HCN4* and *ADCY8*, have also been associated with obsessive-compulsive disorder^39^. Activated cyclic nucleotide-gated channels (HCNs) have been investigated as targets for novel antidepressants^40^, although HCN4 was not one of these cases.

Genes in the bipolar disorder and schizophrenia module (Fig. 2e) were enriched for long-term potentiation (LTP) and circadian entrainment (Fig. 2e). Those unique to bipolar disorder were also enriched for circadian rhythm (PER3 and RORB; Fig. 2e). Insomnia and sleep disorders affect the majority of schizophrenia patients^41^, and are also present in both depressive and manic phases of bipolar disorder^42^. Bipolar disorder patients even show symptoms that are synchronized with the circadian rhythm^43^. Furthermore, genes uniquely connected to schizophrenia were enriched for glutamate receptor transmission and ErbB signaling pathways (Fig. 2e). Neural dysconnectivity, a hallmark of schizophrenia, likely stems from aberrant synaptic plasticity and the incorrect developmental wiring of neurons due to oligodendrocyte malfunction^44^. These processes likely depend most prominently on glutamatergic transmission, neuregulin1 (NRG1)-ErbB signaling, and LTP (refs. ^45,46^). Three schizophrenia-unique genes related to glial cell differentiation were identified (Fig. 2e): ERBB3, PTPRZ1, and SOX10. Indeed, schizophrenia patients present an altered co-expression of genes associated with NFκB signaling along with genes co-expressed in oligodendrocytes, astrocytes, and microglia^16,47^.

The genes in the autism module (Fig. 2f) were enriched for the KEGG term “Inflammatory Bowel Disease (IBD)”. Autism patients have high comorbidity with IBD along with Crohn’s disease^48^. A fecal transplant from healthy subjects, a prospective therapy for IBD (ref. ^49^), has been suggested to alleviate digestive symptoms, and aggressive or repetitive behaviors in some autism patients^50^. The mechanisms behind these effects remain unclear, but the genes related to autism that were found to overlap with those of IBD are associated with inflammatory response, allergy, and the response to helminth parasites (interleukin (IL)6, IL23A, IL13, IL5, HLA-DRB1, HLA-DRB4, IFNG, and TGFB1). Here, IL13 was found to be targeted by drugs that have never been tested for autism. The circadian cycle-related gene RORA, also shared by IBD and autism, perhaps affects immune function due to the disruption of daily rhythms^51^.

### Network medicine framework

**Fig. 3:**
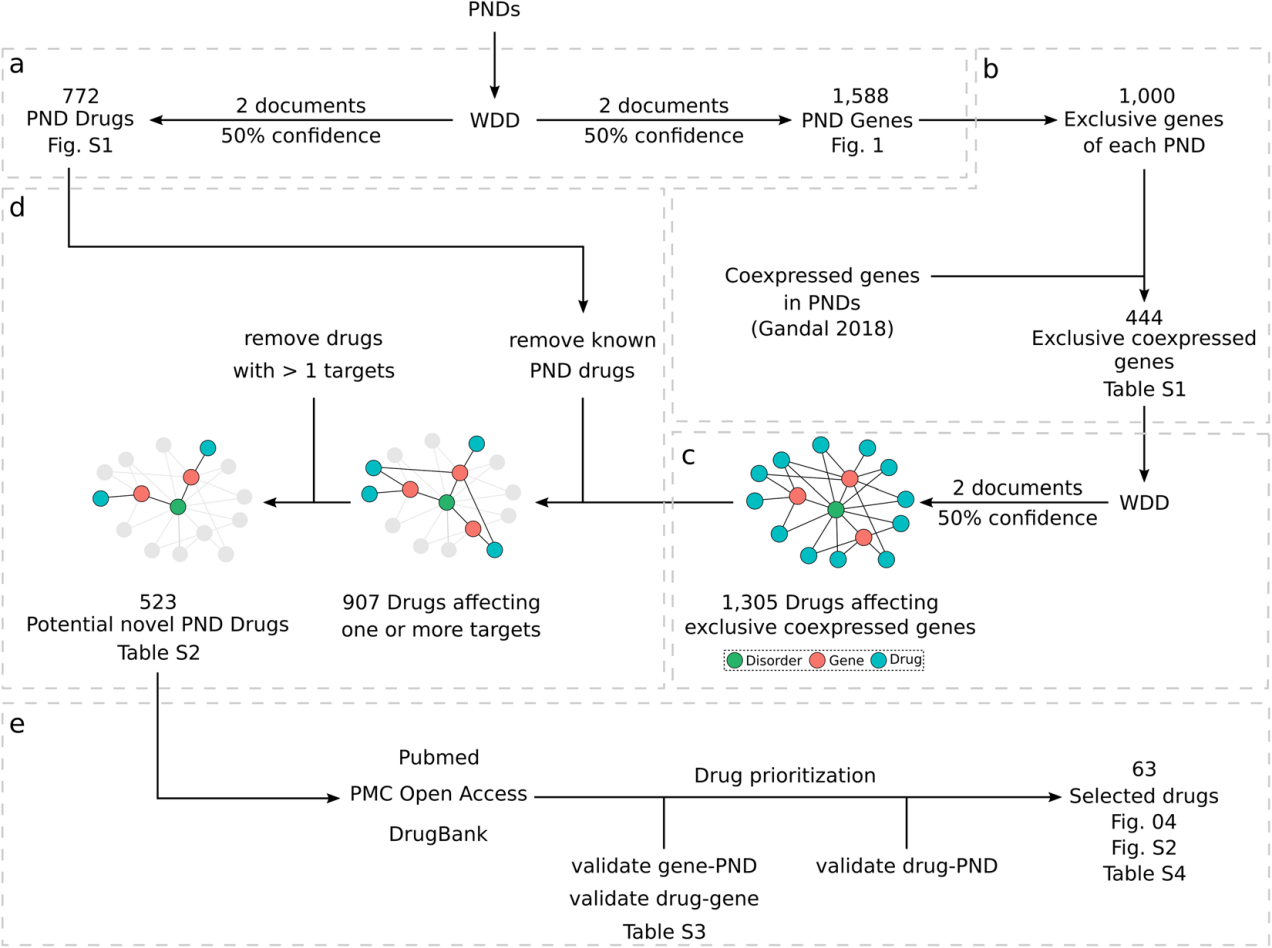
Network medicine framework. **a** WDD searches for gene–PND and drug–PND associations. All WDD results were filtered to keep only relationships supported by two documents or more and at least 50% confidence. **b** Selection of exclusive genes of each PND that are coexpressed in brain tissue according to Gandal et al.^15^. **c** WDD searches for drug–gene associations using exclusive coexpressed genes of PNDs. **d** Removal of drugs obtained in **a** and of drugs targeting more than one gene. **e** Drug prioritization through literature searches and selection of relevant cases.

From the gene–PND network (Fig. 3a), we selected genes that were exclusively associated to each PND and that were coexpressed in brain tissue of PND patients according to Gandal et al.^15^ (Fig. 3b). Co-expression hubs are potential drug targets since they may influence the expression of several other genes^52^. To select the most relevant drugs, we kept only those targeting genes that are co-expressed in brain tissues of PND patients^15^. These co-expression networks included patients and healthy subjects from 700 microarray gene expression studies. The selected genes (Supplementary Table 1) were then submitted to a new round of WDD searches to find drugs associated with them (Fig. 3c). This resulted in 1305 drugs. Since our goal was drug repositioning, we removed from the network 782 drugs known to be associated with the PNDs or that were associated to more than one gene (Fig. 3d, Supplementary Table 2). This allowed a subsequent manual curation of 30% of the results (Fig. 3e). We read each document provided by WDD (Supplementary Table 2) that supports the drug–gene and the gene–PND relationships, and manually removed errors and any relationship that was not in fact described in the documents (examples in Supplementary Table 3). It is important to note that reviewing these 30% of interactions did not constitute a WDD performance evaluation, which was not the goal of our work. With the remaining drugs, we searched the Drug Bank (https://www.drugbank.ca/)^53^ for any ongoing or finished clinical trials, involving these drugs and the PNDs (Fig. 3e). Finally, we performed an open literature review in Pubmed for selected drug–gene–PND relationships (Fig. 3e). We aimed at explaining how each drug could potentially affect their target gene and how this effect could impact the disease. Drugs that presented promising evidence of a viable mechanism that could potentially promote disease altering effects were selected for discussion. The scripts used to perform these steps are available on GitHub (https://github.com/csbl-usp/WDD_drug_repositioning).

### Drug repositioning for PNDs

**Fig. 4:**
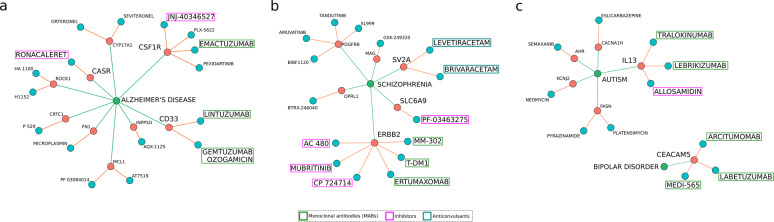
Drugs with high repositioning potential for PNDs. **a**–**c** The most promising repositioning drug candidates (blue) that target the genes (red) unique to Alzheimer’s disease (**a**), schizophrenia (**b**), and autism and bipolar disorder (**c**). The drugs discussed in the main text are highlighted here: monoclonal antibodies (green), inhibitors (magenta), and anticonvulsants (cyan).

Our network medicine framework with WDD was applied to find suitable candidates for drug repositioning for PNDs (Fig. 4, Supplementary Fig. 2). We manually curated the resulting list and selected those drugs with potential for follow-up testing. We prioritized 63 drugs targeting 31 genes and eight PNDs that showed potential for follow-up testing (Supplementary Table 4). Literature search revealed that 18 of those drugs had already been associated with a PND, suggesting that our criteria for initial screening was stringent.

We also used Open Targets^21^ to demonstrate that the drug repositioning framework proposed here can be successfully applied to any drug–gene–disease interaction database (see “Methods”). Applying the same framework described in Fig. 3a–d, we obtained 4670 drugs (Supplementary Table 2). Due to drug name synonyms (which were consolidated in WDD), Open Targets associations include many redundant drug–gene interactions. Nevertheless, we were able to detect 91 drugs in common between WDD and Open Targets (Supplementary Table 5). Promising cases of drugs that target genes connected to Alzheimer’s disease, schizophrenia, bipolar disorder, and autism are depicted in Fig. 4 and described below.

Two anti-CD33 monoclonal antibodies, Gemtuzumab Ozogamicin and Lintuzumab, were identified as promising repositioning candidates concerning Alzheimer’s disease (Fig. 4a). Lintuzumab (also found in Open Targets—Supplementary Table 5) has been reported to reduce the microglial cell surface CD33 by 80% (refs. ^54,55^). CD33 is involved in the inflammatory response related to the amyloid cascade in Alzheimer’s disease^54^. The CD33 gene harbors a single-nucleotide polymorphism (SNP) that protects against Alzheimer’s disease (rs12459419T). This SNP leads to the production of a nonfunctional splicing isoform of CD33, lacking exon 2 (refs. ^54,55^). Treatment with anti-CD33 antibodies might replicate this effect by reducing the CD33 protein levels and reduce microglia-associated neuroinflammation. Drugs targeting the colony stimulation factor 1 receptor (CSF1R) present similar potential (Fig. 4a). This particular gene is essential for microglia survival in the brain^56^. Also, the treatment with CSF1R inhibitors in mice leads to a reduced microglia-mediated accumulation of amyloid plaques^56^ and prevents the development of Alzheimer’s disease-like symptoms due to the anti-inflammatory effect of halting microglia proliferation^57^. We found two drugs, JNJ-40346527 (CSF1R inhibitor) and Emactuzumab (anti-CSF1R monoclonal antibody; Fig. 4a), which have been extensively used in oncology^58^ but never to treat Alzheimer’s disease. Ronacaleret, a calcium-sensing receptor (CASR) inhibitor, could also mitigate the deleterious effects of the amyloid cascade. CASR is expressed by astrocytes and is responsible for detecting synaptic cleft Ca^2+^ concentrations. Extracellular amyloid-β oligomers activate CASR, which induces the accumulation and secretion of more oligomers, nitric oxide release, and VEGF-A expression^59^. This leads to neuronal death, sparing the astrocytes, which continue the amyloid cascade^60^. Halting this pathway early could reduce neuronal death and slow down the progression of Alzheimer’s.

Some schizophrenia-related genes were also promising drug targets (Fig. 4b) found both in WDD and Open Targets (Supplementary Table 5). ERBB2, an essential gene in the NRG1/ErbB signaling pathway, is targeted by six drugs—three inhibitors (AC-480, Mubritinib, and CP 724714) and three monoclonal antibodies (Trastuzumab, Ertumaxomab, and MM-302; Fig. 4b). This pathway is involved in schizophrenia due to its relevance to synaptic transmission and plasticity^45^. Moreover, it is known that NRG1/ErbB signaling triggers myelination in oligodendrocytes^61^. ErbB1 inhibition has been proposed as a potential antipsychotic approach^62^, and the anti-ErB2 monoclonal antibody Trastuzumab has been suggested as a possible treatment tool for schizophrenia^63^. We also found SLC6A9 (glycine transporter 1—GlyT1) inhibitors (Fig. 4b). Bitopertin (NCT01116830) and PF-03463275 (NCT01911676) are actually going through clinical trials for schizophrenia^64,65^ (Fig. 4b). Two anticonvulsant drugs (Levetiracetam and Brivaracetam) targeting the synaptic vesicle glycoprotein 2A are also promising in this respect (Fig. 4b). Levetiracetam (Fig. 4b) was found to improve cognition in a rat model for schizophrenia^66^. Brivaracetam (Fig. 4b) has never been used to treat schizophrenia, which makes it an attractive drug repositioning candidate.

Among the drugs connected to the remaining PND-related genes (Fig. 4c, Supplementary Fig. 2), we highlight those that act upon IL13 for autism and CEACAM5 for bipolar disorder (Fig. 4c). Drugs that affect genes connected to depression, dementia, and Parkinson’s disease are depicted in Supplementary Fig. 2, and discussed in the Supplementary results section. IL13 has increased expression in the T lymphocytes of autism patients^67^ and exerts its inflammatory effects through the acidic mammalian chitinase (AMCase)^68^. In this research, we found two anti-IL13 monoclonal antibodies (Lebrikizumab and Tralokinumab) and an AMCase inhibitor (allosamidin; Fig. 4c). Neither drugs have been tested for autism. CEACAM5 (carcinoembryonic antigen-related cell adhesion molecule 5, OMIM 114890) has been reported to be a potential biomarker for bipolar disorder^69^. CEACAM5 levels are higher in the manic phase^69^, and lithium reduces its levels^70^. Two CEACAM5-specific antibodies and one bi-specific CEA/CD33 antibody with no prior connections to bipolar disorder were also identified using WDD and Open Targets (Fig. 4c, Supplementary Table 5). Although it is unclear whether alterations in CEA serum levels cause or arise due to bipolar disorder, reducing the CEACAM5 serum levels might be a promising approach.

## Discussion

Our results provide robust evidence in favor of Barabási’s shared components hypothesis^13^, which states that “[…]diseases that share disease-associated cellular components (genes, proteins, metabolites, and miRNAs) show phenotypic similarity and comorbidity”. Since we used data obtained entirely from previously published works, none of the individual relationships between genes and drugs, and genes and PNDs, by definition, are novel. Nonetheless, the network medicine framework presented here was able to integrate this accumulated knowledge from the scientific literature to obtain several previously unknown associations between drugs and PNDs. We also showed that this framework can be applied with success to different drug–gene–diseases interaction databases.

PNDs are dimensional conditions with multiple overlapping layers of complexity^71^. We saw that PNDs that share more symptoms, also share more genes. These findings support the idea that PND-related genes are associated with brain networks involved in shared behavioral manifestations, such as cognition and fear-threatening reactions^72^. Our results also confirm the genetic separation between neurological and psychiatric disorders, seen recently using GWAS results^9^.

We were able to break down the inherent characteristics of PNDs to find particularities. Cornerstone biological pathways associated with neuropsychiatry were readily detectable in our data: amyloid beta plaque formation in Alzheimer’s disease and dementia, apoptosis for Parkinsons’s diseases and Hutington disease, synaptic transmission for depression, anxiety, bipolar disorder and schizophrenia, and synaptic organization and cell–cell adhesion for autism (Supplementary results). We were also able to detect consistent characteristics that are just being described in the literature and have not been fully explored yet, such as the involvement of cancer-related miRNAs in Alzheimer’s disease^28^, the regulation of dopamine transmission by the circadian cycle^73^, and the role of subcellular molecular trafficking in Huntington’s disease^32,33^. Our results also supported a genetic relation between depression and heart disease. Hypertrophic cardiomyopathy (HCM) and depression are also common comorbidities^74^, and patients with HCM are correlated with a higher prevalence of depression^75^. Finally, by looking at the complete scope of the literature published in the past 50 years, we were able to identify a consistent neuroimmune/inflammatory genetic signature in all PNDs.

Previous computer-based or experimental drug repositioning frameworks have relied on gene expression, drug–trarget binding or phenotypical screenings to find candidates^76,77^. WDD does not distinguish associations between genes and disorders that occur due to SNPs, epigenetic modifications, or differences in expression. If two PNDs share a gene, each relation could involve a different mechanism. No information on drug effect direction is provided either. Thus, we had to compromise in selecting potential candidates for drug repositioning; we decided to only keep drugs that were found to affect one PND gene. This reduced the amount of collected data, which allowed us to validate several drug–gene–PND connection individually and prioritize candidates for discussion. There was a caveat of increasing specificity and decreasing the potential reach of the drugs. Since PNDs are complex, omnigenic disorders^71^, solutions focused on individual genes may not be ideal. We selected drugs affecting genes that are coexpressed in brain tissue from PND patients^15^. Co-expressed genes usually play more critical roles in diseases, are more often targeted by drugs, and have influence over the expression of other genes^52^. We predict that the reach of the selected drugs will be broad due to the coexpressed nature of their targets. Using this approach, we found monoclonal antibodies with repositioning potential. Monoclonal antibodies are highly specific concerning their targets, but they are also large molecules with low permeability through the blood–brain barrier. This can be a challenge in the follow-up validation of the drugs found in this research. However, some of these antibodies are being discussed as viable alternatives for treatment of schizophrenia and Alzheimer’s disease^54,63^, which indicates that our approach is efficient in finding potential candidates for drug repositioning. We also found drugs that could be used in combination to treat PNDs. Six drugs that could treat schizophrenia were found to target ERBB2. Recently, the NRG1-ERBB4 signaling antagonist Spironolactone was identified in a cell-based drug repositioning screening as a candidate for clinical trials for schizophrenia^77^. These drugs have the potential to reduce dysconnectivity and hallucination by regulating the NRG1-ErbB signaling. Four drugs could be used synergistically to reduce microglia-mediated inflammation in Alzheimer’s disease, through the inhibition of CSF1R and CD33. Three other drugs were also found to target IL13, which could be used to reduce the inflammatory response in autism.

Our network medicine approach was able to successfully integrate the data obtained from millions of scientific papers using complex networks to generate new insights about PNDs. The network medicine framework proposed here can be applied to drug–gene–disease interaction databases, such as WDD and Open Targets. The drugs we selected here are highly promising candidates for repositioning that could be taken into consideration for follow-up in vitro and in vivo screenings.

## Supplementary information

Supplementary results

Figure S1

Figure S2

Table S1

Table S2

Table S3

Table S4

Table S5
