# Supplementary material for: Drug repositioning for psychiatric and neurological disorders through a network medicine approach
Source: Transl Psychiatry. 2020 May 12;10:141. doi: 10.1038/s41398-020-0827-5 (PMC7217930; doi:10.1038/s41398-020-0827-5)
Supplement: Supplementary file 1 — Supplementary results [file 41398_2020_827_MOESM1_ESM.docx]

**Supplementary Material**

**Results**

**Drug-PND Knowledge Network**

The drug-PND network showed a high overlap between PNDs (Fig. S1a). The distinction between neurodegenerative and psychiatric disorders seen for genes was less clear in the drug network (Fig. S1a). Autism, for instance, was located closer to schizophrenia in the gene network, but closer to Parkinson’s disease in the drug network (Fig. S1a). Also, PNDs that clustered together in the gene network (Fig. 1a) such as schizophrenia and bipolar disorder, were not placed in the same module in the drug network (Fig. S1a). Furthermore, out of 36 PND combinations, 32 pairs (90%) showed significant drug overlap (Fig. S1b). In contrast, only 19 PND-pairs (53%) significantly shared genes with each other (Fig. 1b).

**Functional enrichment**

In each PND module, we found several well-established, hallmark molecular mechanisms of psychiatric and neurological disorders. These findings are depicted in Figure 2a and described bellow.

The genes in the Alzheimer’s disease and dementia module enriched for amyloid beta plaque formation and apoptosis, the hallmark mechanisms in these disorders. Genes in this module also enriched for phospholipid homeostasis and regulation of phosphorylation. Phospholipid dysregulation is involved in Tau hyperphosphorylation following traumatic brain injury^1^⁠, a cause of dementia not related to Alzheimer’s disease. Huntington’s and Parkinson’s disease genes enriched for regulation of apoptosis, response to oxidative stress and regulation of autophagy, processes related to cell-death and degeneration in these disorders. The genes in the depression and anxiety module enriched for serotonergic synapse. Selective serotonin reuptake inhibitors are used to treat these disorders, which present decreased synaptic serotonin. Enrichment in the bipolar disorder/schizophrenia module was seen for cocaine and amphetamine addiction. The relation between these disorders and dopamine dysregulation in the reward anticipation activity, also seen in drug addiction, is well established. Autism genes enriched for synaptic organization and cell-cell adhesion. Autism is indeed thought to be a result of long range hypoconnectivity in the brain.

**Drug repositioning**

Among the drugs targeting genes connected to depression, Parkinson’s disease and dementia, we highlight the following.

Inclacumab (Fig. S2a) is a P-selectin (*SELP*) inhibitor^2^⁠. P-selectin levels are significantly increased in depression patients^3^⁠, which is related to the elevated inflammatory state seen in this disorder. SIAH1 has a potential role in Parkinson’s disease due to its presence in Lewy bodies and its role in apoptosis^4^⁠. Despite no disease risk increasing mutation has been found in this gene^4^⁠, it has been shown to be a co-expression hub in PNDs^5^⁠, which makes it a suitable candidate for manipulation. Here we found a drug, Omigapil (Fig. S1b), a drug that prevents GAPDH-SIAH1 mediated apoptosis^6^⁠, which has been tested for Parkinson’s disease with conflicting results^7,8^⁠. Resistin (*RETN*) has been show to increase LDL levels, which increases heart disease risk. Here we found that Ginkgolide A (Fig. S1c) inhibits resistin induced endothelial permeability^9^⁠. This drug could, therefore, potentially reduce the resistin associated vascular risk of dementia^10^⁠.

**References**

1 Cao J, Gaamouch F El, Meabon JS, Meeker KD, Zhu L, Zhong MB *et al.* ApoE4-associated phospholipid dysregulation contributes to development of Tau hyper-phosphorylation after traumatic brain injury. *Sci Rep* 2017; **7**: 11372.

2 Stähli BE, Gebhard C, Duchatelle V, Cournoyer D, Petroni T, Tanguay JF *et al.* Effects of the P-Selectin Antagonist Inclacumab on Myocardial Damage After Percutaneous Coronary Intervention According to Timing of Infusion: Insights from the SELECT-ACS Trial. *J Am Heart Assoc* 2016; **5**: 1–8.

3 Neubauer H, Petrak F, Zahn D, Pepinghege F, Hägele AK, Pirkl PA *et al.* Newly diagnosed depression is associated with increased beta-thromboglobulin levels and increased expression of platelet activation markers and platelet derived CD40-CD40L. *J Psychiatr Res* 2013; **47**: 865–871.

4 Franck T, Krueger R, Woitalla D, Müller T, Engelender S, Riess O. Mutation analysis of the seven in absentia homolog 1 (SIAH1) gene in Parkinson’s disease. *J Neural Transm* 2006; **113**: 1903–1908.

5 Gandal MJ, Haney JR, Parikshak NN, Leppa V, Ramaswami G, Hartl C *et al.* Shared molecular neuropathology across major psychiatric disorders parallels polygenic overlap. *Science (80- )* 2018; **359**: 693–697.

6 Erb M, Meinen S, Barzaghi P, Sumanovski LT, Courdier-Früh I, Rüegg MA *et al.* Omigapil ameliorates the pathology of muscle dystrophy caused by laminin-α2 deficiency. *J Pharmacol Exp Ther* 2009; **331**: 787–795.

7 Olanow CW, Schapira AH, LeWitt PA, Kieburtz K, Sauer D, Olivieri G *et al.* TCH346 as a neuroprotective drug in Parkinson’s disease: a double-blind, randomised, controlled trial. *Lancet Neurol* 2006; **5**: 1013–1020.

8 Andringa G, Cools AR. The neuroprotective effects of CGP 3466B in the best in vivo model of Parkinson’s disease, the bilaterally MPTP-treated rhesus monkey. *J Neural Transm Suppl* 2000; : 215–225.

9 Jamaluddin MS, Yan S, Lü J, Liang Z, Yao Q, Chen C. Resistin increases monolayer permeability of human coronary artery endothelial cells. *PLoS One* 2013; **8**. doi:10.1371/journal.pone.0084576.

10 Bednarska-Makaruk M, Graban A, Wiśniewska A, Łojkowska W, Bochyńska A, Gugała-Iwaniuk M *et al.* Association of adiponectin, leptin and resistin with inflammatory markers and obesity in dementia. *Biogerontology* 2017; **18**: 561–580.

**Fig. S1. Drugs shared between PNDs. a** knowledge network for drugs colored according to Louvain-defined modules: Alzheimer’s disease (AD) and dementia (DM) (purple), Huntington’s disease (HD) and Parkinson’s disease (PKD) (dark blue), depression (MDD) and anxiety (AX) (red), schizophrenia (SCZ) (green-yellow) and bipolar disorder (BD) (yellow) and autism (ASD) (green). **b** Drug overlap significance between PNDs. Larger nodes represent PNDs with more drugs and thicker edges represent more significant overlap between PNDs (proportional to -log_10_p-value of the Fisher’s Exact test).

**Fig. S2. Drugs with high repositioning positioning for PNDs not discussed in the main text. a-c** Most promising repositioning drug candidates (blue) that target unique genes (red) of depression (**a**), Parkinson’s disease (**b**) and dementia (**c**).

**Table S1. Genes used in second round searches (WDD and Open Targets). Tab 1:** WDD exclusive coexpressed genes. Source: genes. Target: PNDs. Confidence: WDD score. Document IDs: identification of papers supporting the relationship (Medline=Pubmed, Pmcoa=PMC Open Access). **Tab 2:** Open Targets exclusive coexpressed genes. Source: genes. Target: PNDs. score: Open Targets score.

**Table S2. Potential novel PND drugs (WDD and Open Targets). Tab 1:** potential novel drugs found using WDD following the steps in Fig. 3a-d. Drug: potential drugs. Gene: genes affected by the potential novel drugs. gene_PND_ref: references of the relationships between genes and PNDs provided by WDD. drug_gene_ref: references of the relationships between drugs and genes provided by WDD. **Tab 2:** potential novel drugs found using WDD that were submitted to drug prioritization. PND: PND associated with the genes. DRUG: potential drugs. GENE: genes affected by the selected potential novel drugs. **Tab 3:** potential novel drugs found using Open Targets following the steps in Fig. 3a-d. Drug: potential drugs. Gene: genes affected by the potential novel drugs.

**Table S3. Manual curation examples. Tab 1:** false gene-PND associations found by WDD. Disease: PNDs. Gene: genes falsely associated with the PNDs. WDD Reference: references of the relationships between genes and PNDs provided by WDD. Sentence: sentences extracted from references provided by WDD that show that the gene-PND associations are false. Error: reason why the gene-PND associations are false. **Tab 2:** false drug-gene associations found by WDD. Disease: PNDs. Gene: genes associated with the PNDs. Drug: drugs falsely associated with the genes. WDD reference: references of the relationships between drugs and genes provided by WDD. Sentence: sentences extracted from references provided by WDD that show that the drug-gene associations are false. Error: reason why the drug-gene associations are false.

**Table S4. Prioritized drug candidates selected with the network medicine framework.** Disease: PNDs. Drug: prioritized drug candidates. Comercial/alternative name: alternative drug names. Target gene: genes targeted by the prioritized drug candidates. Drug effect: how the prioritized drug candidates affect the gene. Previous association with PND: information on whether there was previous association of the drug with any PND in the literature. Gene-disease references: references of the relationships between genes and PNDs provided by WDD. Gene-disease reference example sentence: sentences extracted from the references provided by WDD that show that the drug-gene associations are true. Drug-gene references: references of the relationships between drugs and genes provided by WDD.

**Table S5. Comparison of the network medicine framework between WDD and Open Targets. Tab 1:** number of genes and drugs found after each step of the network medicine framework in WDD or Open Targets and in common between the two. **Tab 2:** drugs found both in WDD and Open Targets (after steps in Fig 3a-d).
