# Supplementary figures and images for: Drug repositioning for psychiatric and neurological disorders through a network medicine approach
Source: Transl Psychiatry. 2020 May 12;10:141. doi: 10.1038/s41398-020-0827-5 (PMC7217930; doi:10.1038/s41398-020-0827-5)

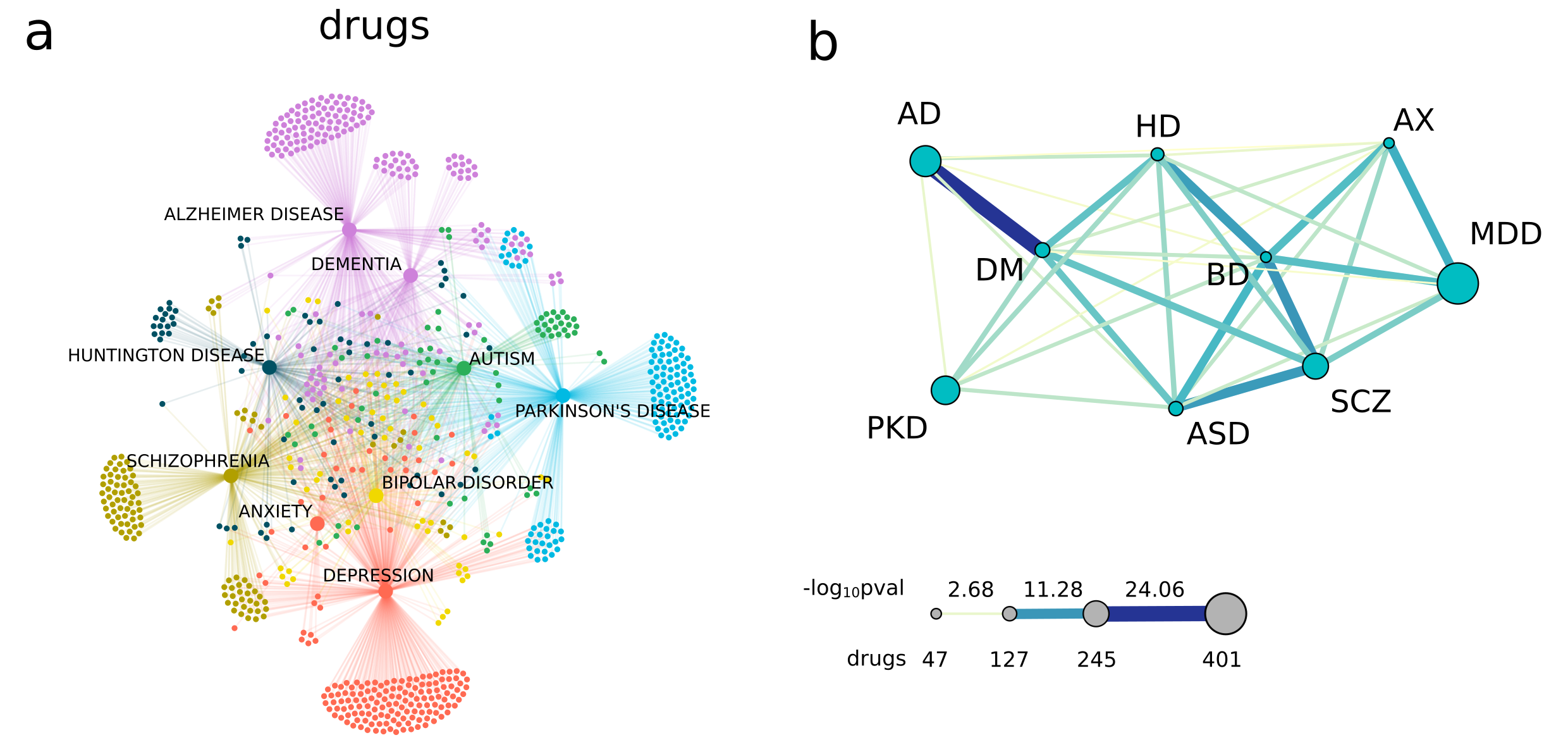

Supplement: Supplementary file 2 — Figure S1 [file 41398_2020_827_MOESM2_ESM.tif]

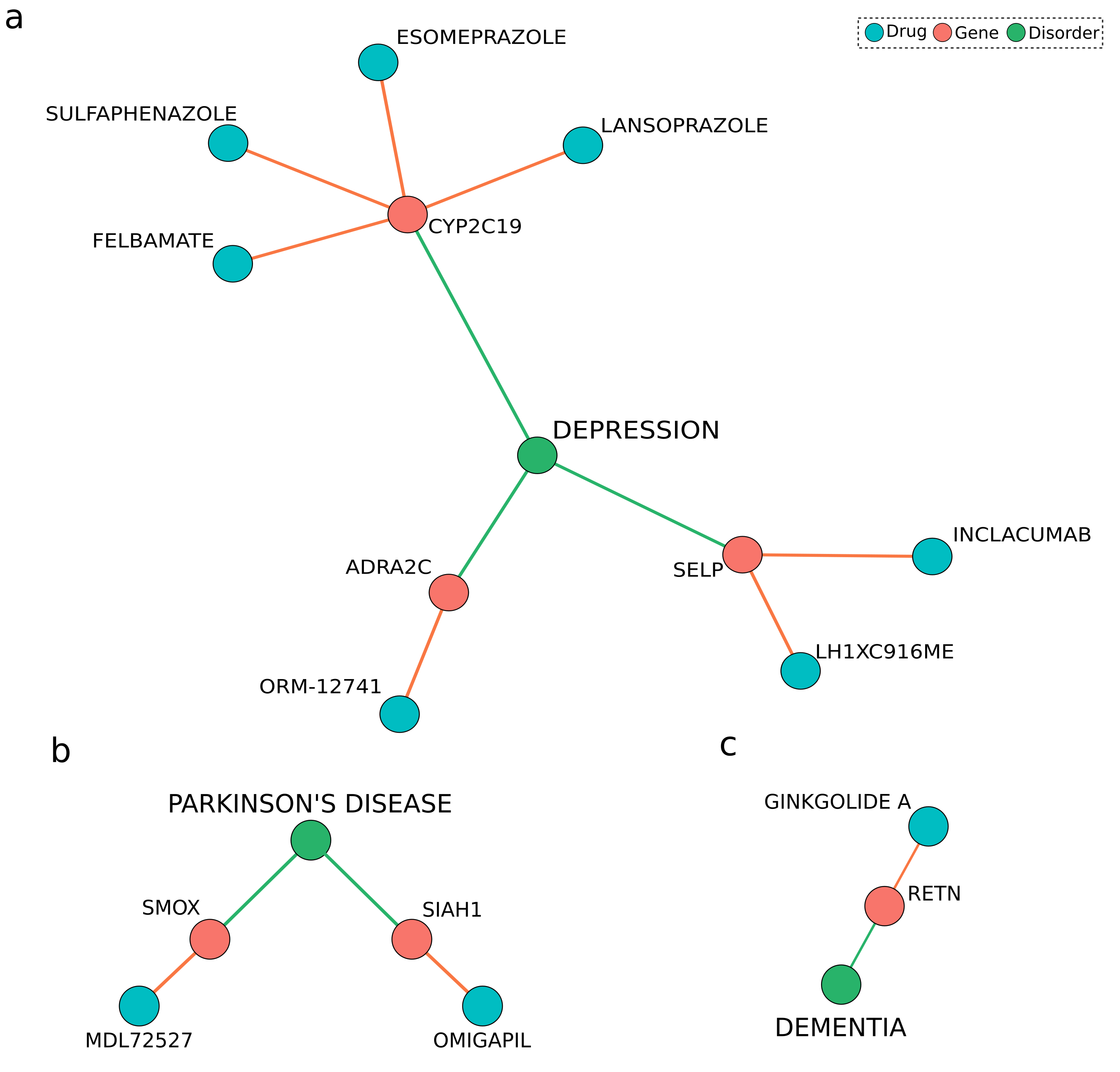

Supplement: Supplementary file 3 — Figure S2 [file 41398_2020_827_MOESM3_ESM.tif]
